# Skeletal Muscle Remodelling as a Function of Disease Progression in Amyotrophic Lateral Sclerosis

**DOI:** 10.1155/2016/5930621

**Published:** 2016-04-18

**Authors:** L. Jensen, L. H. Jørgensen, R. D. Bech, U. Frandsen, H. D. Schrøder

**Affiliations:** ^1^Institute of Clinical Research, Clinical Pathology, SDU Muscle Research Cluster, University of Southern Denmark and Odense University Hospital, J. B. Winsløw Vej 15, 3, 5000 Odense C, Denmark; ^2^Department of Sports Science and Clinical Biomechanics, SDU Muscle Research Cluster, University of Southern Denmark, Campusvej 55, 5230 Odense M, Denmark; ^3^Institute of Clinical Research, The Orthopaedic Research Unit, University of Southern Denmark and Odense University Hospital, Sdr. Boulevard 29, 5000 Odense C, Denmark

## Abstract

Muscle weakness is considered the pivotal sign of amyotrophic lateral sclerosis (ALS). Knowledge about the skeletal muscle degeneration/regeneration process and the myogenic potential is limited in ALS patients. Therefore, we investigate these processes in a time course perspective by analysing skeletal muscle biopsies from ALS patients collected before and after a 12-week period of normal daily activities and compare these with healthy age-matched control tissue. We do this by evaluating mRNA and protein (immunohistochemical) markers of regeneration, neurodegeneration, myogenesis, cell cycle regulation, and inflammation. Our results show morphological changes indicative of active denervation and reinnervation and an increase in small atrophic fibres. We demonstrate differences between ALS and controls in pathways controlling skeletal muscle homeostasis, cytoskeletal and regenerative markers, neurodegenerative factors, myogenic factors, cell cycle determinants, and inflammatory markers. Our results on Pax7 and MyoD protein expression suggest that proliferation and differentiation of skeletal muscle stem cells are affected in ALS patients, and the myogenic processes cannot overcome the denervation-induced wasting.

## 1. Introduction

Amyotrophic lateral sclerosis (ALS), also known as motor neuron (MN) disease, is the most common motor neuron disorder among adults [1]. ALS is characterized by degeneration of upper motor neurons leading to spasticity and lower motor neurons resulting in interruption of the nerve connection at the neuromuscular junction (NMJ) and myofibre loss [2, 3]. The affected neuromuscular system attempts to restore function by nerve sprouting for reinnervation and by myogenesis to regenerate the muscle fibres [4]. Eventually, persistent muscle wasting exceeds the ability to repair and the consequence is progressive muscle atrophy, respiratory insufficiency, and usually death within 3–5 years of diagnosis [5]. Knowledge about the skeletal muscle degeneration/regeneration process and the myogenic potential is limited in ALS patients and has, to our knowledge, never been investigated in a time course perspective.

Muscle weakness is considered the cardinal sign of ALS and debate still exists as to whether denervation originates from the neuron or the muscle [4]. Much of our current knowledge about ALS disease progression is established within the superoxide dismutase (SOD1) G93A mouse model [6]. Previous studies show that muscle specific expression of mutant SOD1 in mice leads to an ALS phenotype and degradation of MNs [7, 8], demonstrating that abnormalities in skeletal muscle can induce degradation of MNs and cause ALS symptoms.

Human ALS samples are rare, but autopsy of an ALS patient demonstrated skeletal muscle changes with clear signs of denervation and reinnervation. However, the patient had normal appearing motor neurons; thus pathological changes in skeletal muscle appear to be present before the motor neurons are affected [2], thus providing human evidence for the “dying-back” phenomenon.

Healthy skeletal muscle has a remarkable ability to regenerate after injury and tissue damage. The skeletal muscle stem cells, the satellite cells (SCs), that reside under the basal lamina of muscle fibres are responsible for this repair [9]. Upon activation, the SCs become myoblasts and proliferate and differentiate to restore and replace damaged muscle fibres [10].* In vitro* cultures of SCs from ALS patients demonstrate a senescent-like morphology, disturbed differentiation, and an apparent inability to proceed through the myogenic program [11, 12] resulting in a decreased ability to regenerate and mature to functional myofibres.

Together, these findings suggest an important role of skeletal muscle homeostasis, metabolic condition, and regenerative ability in the development of ALS. To slow down disease progression, maintenance of skeletal muscle function may be the most promising therapeutic target as of today. Thus, improved knowledge about the skeletal muscle degeneration/regeneration process is warranted, particularly in a time course perspective.

Therefore, in the present study we describe the degenerative and regenerative processes of skeletal muscle of human ALS patients and how different markers change during 12 weeks of disease progression. The hypothesis is that the myogenic program is ineffective in patients with ALS compared with healthy age-matched subjects.

## 2. Materials and Methods

**Figure 1:**
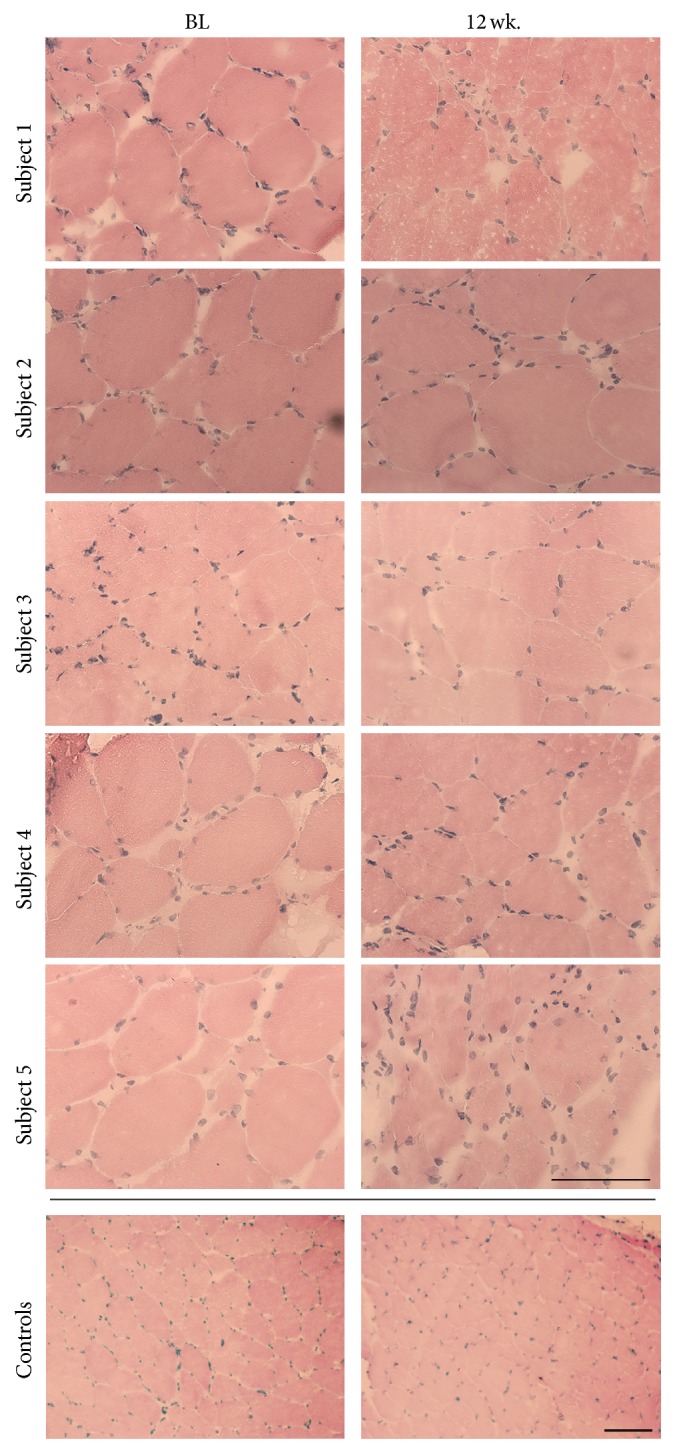
Morphological changes induced during 12 weeks of disease progression. Hematoxylin and eosin (HE) stainings of skeletal muscle biopsies obtained at baseline (BL) and after 12 weeks (wk.). Control samples show representative images of two healthy age-matched muscles. *n* = 5 for ALS and *n* = 5 for controls. Scale bar = 100 *μ*m.

Five subjects clinically diagnosed with ALS (aged 61 ± 2.7 yrs) agreed to participate in this study. The patients were diagnosed at the Department of Neurology, Odense University Hospital according to the El Escorial criteria [13]. Four of them were diagnosed within one year from inclusion, whereas the last one was diagnosed 15 years earlier (subject 4, Figure 1). Inclusion criteria were definite or probable ALS, being ambulatory at onset of study, and willingness to comply with the study protocol. Patients were excluded for other neurological or serious medical problems including cardiovascular disease and diabetes mellitus. See Table 1 for additional clinical details. All subjects were evaluated using the revised ALS functional rating scale (ALSFRS-R) [14], which showed that all patients presented with a similar functional score at the time of inclusion and their disease symptoms showed a comparable decline over the 12-week period (Table 1), thus allowing us to group the patients for all analyses. The participants were informed about the purpose of the study and gave written informed consent before their participation. The study was conducted according to the Declaration of Helsinki and was approved by the Local Ethics Committee of the Region of Southern Denmark, Denmark.

### 2.1. Sample Collection

Muscle biopsies were obtained at baseline (BL) and after 12 weeks (12 wk.). They were collected from* m. vastus lateralis* under local anaesthetic (1% lidocaine; Amgros, Copenhagen, Denmark) using a 5 mm Bergström needle. Biopsies were taken in the same region and depth of the weakest leg and care was taken to avoid any damaging effect of multiple biopsies [15] by separating incisions by about 5 cm. Fifty to hundred mg tissue was excised and divided into multiple pieces. These were either snap-frozen in liquid nitrogen or embedded in Tissue-Tek (Sakura Finetek, Alphen aan den Rijn, Netherlands) and frozen in precooled isopentane. The samples were stored at −80°C for further analysis. Furthermore, muscle biopsies were collected form healthy aged-matched control subjects (*n* = 7, aged 65.1 ± 0.8 yrs.) using the above protocol.

### 2.2. RNA Extraction and cDNA Synthesis

Tissue samples were homogenized and total RNA was extracted according to the manufacturers directions including a salt wash [16] and RNA concentration was measured using the NanoDrop (ND1000, Thermo Scientific, Copenhagen, Denmark) returning 260/280 ratios above 1.8 for all samples, and intact RNA was confirmed by denaturing agarose gel electrophoresis (Bioanalyzer; Agilent Technologies, Glostrup, Denmark). 500 ng of RNA was converted into complementary DNA using High Capacity cDNA Reverse Transcription Kit (Applied Biosystems, Copenhagen, Denmark).

### 2.3. Real-Time Reverse Transcriptase Polymerase Chain Reaction

Real-time RT-PCR for ACTA1, CHRNA1, GDNF, CDK5, MYOG, MYOD1, NCAM, CD68, and PTPRC/CD45 was performed using TaqMan Low Density Arrays. Each port on the card was loaded with cDNA equivalent to 125 ng total RNA and run at 50 cycles in triplicate on the 7900 Sequence Detection System (all reagents from Applied Biosystems, Copenhagen, Denmark). For MYF5, PAX7, P21, P27, SMAD3, and IGF1 real-time RT-PCR was performed in reactions of 25 *μ*L by mixing 6.25 ng cDNA with appropriate TaqMan primers/probes and run in triplicate as described above. Specific primer details can be found in Table S1 “(see Supplementary Material available online at http://dx.doi.org/10.1155/2016/5930621)”. Data was collected and analysed using SDS 2.4 or QuantStudio 12K software (Applied Biosystems). Technical triplicates were evaluated and samples excluded when ΔCt > 0.5. Control genes were verified using geNorm software and data were normalized to RPLP0 and GAPDH using the qBase+ software (Biogazelle, Zwijnaarde, Belgium) [17]. Data are scaled and presented relative to the same control sample (=1) for all mRNA analyses: *n* = 5 for ALS patients and *n* = 7 for controls.

### 2.4. MicroRNA Analysis

Thirty *μ*g total RNA was reverse transcribed using a TaqMan MicroRNA Reverse Transcription Kit (4366596) and RT primers specific for miR-1, miR-27b, mir-133a, miR-26a, miR-206, miR-29b, miR-23a, miR-455, and controls RNU24 and RNU6b according to manufacturers instructions. The RT products were then amplified in triplicate using sequence complementary TaqMan Small RNA assays. All reagents were from Applied Biosystems. Data was collected and analysed using SDS 2.4 and normalized to RNU24 using the qBase+ software (Biogazelle, Zwijnaarde, Belgium): *n* = 5 for ALS and *n* = 7 for controls.

### 2.5. Immunohistochemistry and Quantifications

Ten *μ*m sections of frozen tissue were fixed, permeabilised, and blocked before they were incubated with primary antibodies (1 h, at room temperature). Primary antibodies include MHC-slow 1 : 2000 (M8421, Sigma-Aldrich, Copenhagen, Denmark), MHC-fast 1 : 2000 (M4276, Sigma-Aldrich), MyoD1 1 : 25 (386R-16, Cell Marque/Sigma-Aldrich), Pax7 1 : 100 (Hybridoma Bank, Iowa City, USA), Ki67 1 : 100 (MiB-1, Dako), NCAM 1 : 100 (Leu19/MY31, BD Biosciences, Albertslund, Denmark), and CD68 1 : 100 (M0814, Dako). Then, ImmPRESS AP Ig polymer detection kit and Vector Blue or Vector Red AP Substrate Kit (Vector Laboratories, Herlev, Denmark), Envision+/DAB+ (K4001, K4003, and K3468, Dako, Copenhagen, Denmark), or Alexa Fluor 488 or 555 anti-mouse or anti-rabbit, accordingly 1 : 500 (Thermo Fisher Scientific, Denmark), were applied according to manufacturers protocols. Immunofluorescence stainings were mounted with ProLong Diamond Antifade Mountant with DAPI (Thermo Fisher Scientific). Digital image processing and analyses were performed using Axio imager M1 and AxioVision by Zeiss (Brock & Michelsen, Copenhagen, Denmark).

In all quantifications the investigator was blinded to the identity of the patients. Cross-sectional area (CSA) of type I and type II muscle fibres was determined, and only truly transverse cut fibres were included in the analysis. The percentage of fibres belonging to every CSA interval was determined for each patient before calculating the mean. Furthermore, Pax7^+^ cells, Pax7^+^/MyoD^−^ cells, Pax7^+^/MyoD^+^ cells, MyoD^+^/Ki67^+^ cells, NCAM^+^ cells, and NCAM^+^ fibres were determined and related to the total number of fibres. At each time point *n* = 5 for ALS samples and *n* = 3–5 for controls due to limited control sample tissue.

### 2.6. Statistical Analysis

Data are presented as mean ± SEM, apart from mRNA expression data, which were logarithmically transformed before statistical analysis and are reported in the paper as geometric means ± SEM (back-transformed values). Paired and unpaired *t*-tests were used for statistical analyses (GraphPad Software, version 6.0, La Jolla, USA) of differences between BL and 12 wk. and BL or 12 wk. and CON for mRNA, miRNA, and immunohistochemistry stainings. Frequency distributions for CSA between BL and 12 wk. were compared using Fisher's LSD Test. Data was considered significant when *p* < 0.05. Asterisk indicates significant difference from controls; ^*∗*^ *p* < 0.05, ^*∗∗*^ *p* < 0.01, and ^*∗∗∗*^ *p* < 0.001, while # indicates significant difference between BL and 12 wk. in ALS patients. () indicates a tendency for a significant difference (*p* < 0.1).

## 3. Results

### 3.1. Visible Morphological Changes in ALS

Figure 1 shows HE stainings from ALS subjects at BL and 12 wk. and from two control samples and reveals morphological changes with increasingly disorganised muscle fibres including increased number of atrophic fibres and compensatory hypertrophy in all ALS biopsies. Subjects 2 and 5 are particularly affected by disease progression demonstrating a high degree of atrophy, compensatory hypertrophy, and areas of macrophage infiltration after 12 wk., while the remaining subjects present with milder morphological changes including the same features. Furthermore, increased amounts of inflammatory cells as well as nuclear clumps are present between the muscle fibres at 12 weeks compared to BL.

**Figure 2:**
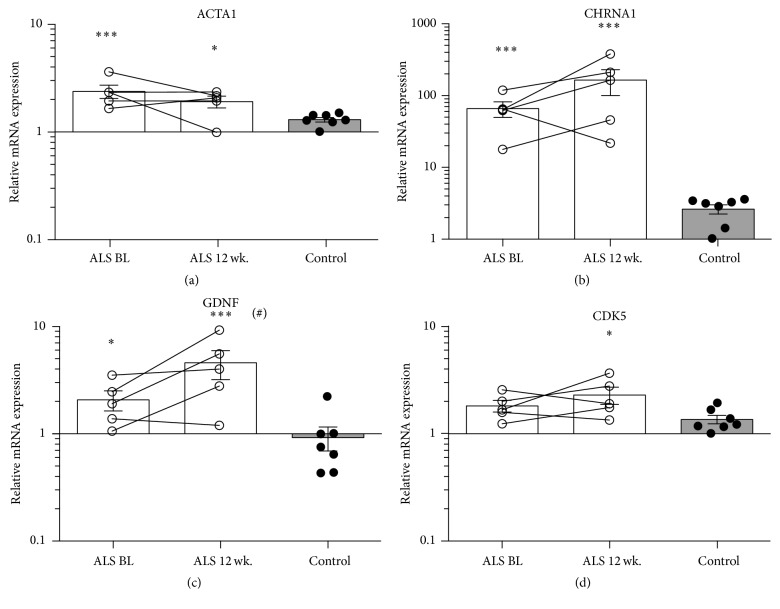
Evidence of skeletal muscle remodelling and motor neuron denervation. mRNA levels of *α*-actin (ACTA1) (a), acetylcholine receptor (CHRNA1) (b), glial derived neurotropic factor (GDNF) (c), and cyclin-dependent kinase 5 (CDK5) (d) at BL and 12 wk. compared to age-matched controls (^#^ *p* < 0.1 compared to BL). Asterisks indicate significant difference from controls; ^*∗*^ *p* < 0.05 and ^*∗∗∗*^ *p* < 0.001.

The morphological changes are supported by an increase in *α*-actin (ACTA1), since *α*-actin is known to be induced during active remodelling, for example, exercise and regeneration [18, 19]. Furthermore, denervation is evident by increases in the nerve and NMJ promoting factors acetylcholine receptor (CHRNA1), glial derived neurotropic factor (GDNF), and cyclin-dependent kinase 5 (CDK5), when compared to controls (Figure 2).

### 3.2. Changes in Fibre Type Distribution and Fibre Cross-Sectional Area

Baseline and 12 wk. biopsies demonstrate individual disease-induced changes in fibre type distribution and mean CSA, but none of the changes are significant at a group level (Table 2).

**Figure 3:**
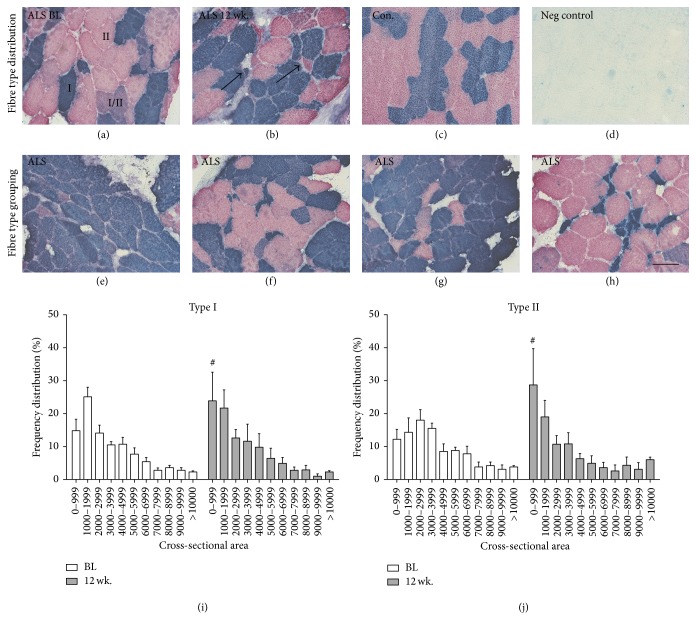
Skeletal muscle of ALS patients presents with atrophy of both type I and type II fibres. (a and b) Representative images of immunohistochemical stainings for fibre type distribution show MHC-slow in blue, MHC-fast in red, and mixed/changing fibres in purple. Atrophic fibres of both type I and type II can be seen in the same individual (b, arrow). (c) Control samples show even distribution of fibres with similar size and no mixed fibres. (e–h) Representative images of fibre type grouping and fibre type-specific atrophy in ALS muscle. (I and j) Frequency distribution of cross-sectional area at BL and 12 wk. for type I (i) and type II (j) fibres. CSA: cross-sectional area. Significant differences between BL and 12 wk. in ALS in CSA for both fibre types are indicated by ^#^ *p* < 0.05. *n* = 5 for ALS and *n* = 3 for controls. Scale bar = 50 *μ*m.

Immunohistochemical stainings for MHC-slow (type I fibres) and MHC-fast (type II fibres) demonstrate atrophy and compensatory hypertrophy of both fibre types (Figures 3(a) and 3(b)), as well as areas with fibre type grouping dominated by either type I fibres or type II fibres (Figures 3(e)–3(h)). The distribution of individual fibre CSA displays large variability in fibre size with fibres raging from below 1000 *μ*m^2^ to more than 10.000 *μ*m^2^ (Figures 3(i) and 3(j)). When comparing the frequency distributions of CSA at BL and 12 wk. there is a shift towards a smaller fibre size after 12 wk. in both type I and type II fibres (*p* < 0.05); thus ALS muscle contains a larger number of atrophic fibres at 12 wk. compared to BL.

### 3.3. Myogenic Changes in ALS

**Figure 4:**
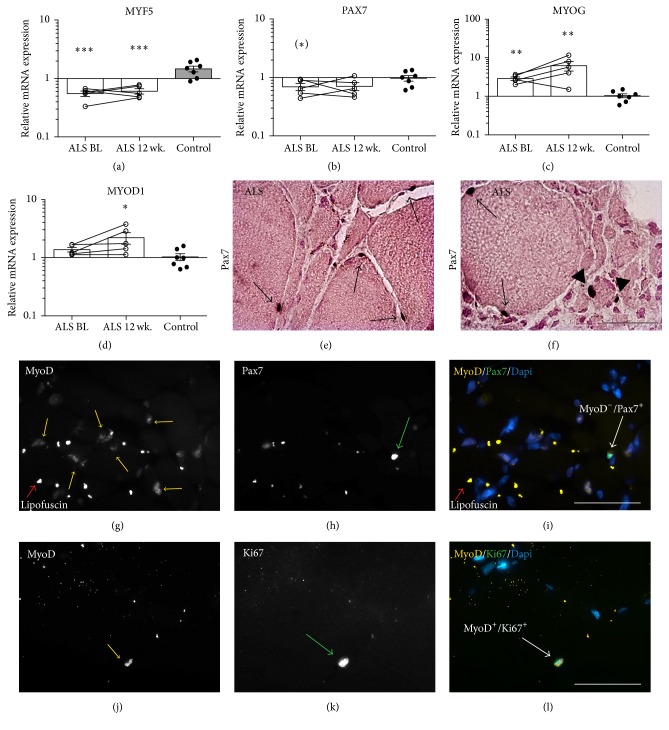
Myogenesis in ALS muscle. mRNA transcripts of (a) MYF5, (b) PAX7, (c) MYOG, and (d) MYOD1 in ALS during disease progression compared to controls. (e and f) Representative immunohistochemical images of Pax7^+^ cells in ALS muscle. Arrows indicate Pax7^+^ cells associated with hypertrophied fibres and arrowheads indicate Pax7^+^ cells associated with atrophic fibres. (g–i) Double immunofluorescence stainings of MyoD (yellow), Pax7 (green), and DAPI (blue). (j–l) Double immunofluorescence stainings of MyoD (yellow), Ki67 (green), and DAPI (blue). (^∗^) *p* < 0.1, ^*∗*^ *p* < 0.05, ^*∗∗*^ *p* < 0.01, and ^*∗∗∗*^ *p* < 0.001. In (a–d) *n* = 5 for ALS and *n* = 7 for controls and in (g–l) *n* = 5 for ALS. Scale bar = 50 *μ*m for all images.

Skeletal muscle of ALS patients shows a reduction in mRNA levels of satellite cell and early myogenic markers PAX7 and MYF5 compared to controls, whereas the mRNA levels of the late myogenic markers myogenin (MYOG) and MYOD1 are increased (Figures 4(a)–4(d)). The mRNA levels of MYOG and MYOD1 are increased at 12 wk. compared to BL, although this does not reach statistical significance.

The mRNA results suggest that the satellite cells might be affected in the ALS patients. We therefore analysed the activation status of the satellite cells using immunohistochemistry. Pax7^+^ cells were found next to both hypertrophied fibres (Figures 4(e) and 4(f), arrows) and in smaller fibres (Figure 4(f), arrowhead). The overall number of Pax7^+^ cells/fibre did not change between BL and 12 wk. (Table 3). To investigate whether the satellite cells are activated or are resting we performed double immunofluorescence staining for Pax7 and MyoD. We find that that very few Pax7^+^ cells express MyoD (Pax7^+^/MyoD^+^); thus the majority of the Pax7^+^ satellite cells are negative for MyoD (Table 3 and Figures 4(g)–4(i)). On an interesting note, we observed a large amount of MyoD positive nuclei in the tissue in general for all patients (Figures 4(g) and 4(j)). To evaluate whether these MyoD^+^ cells were in an active proliferative state or postmitotic, we performed a Ki67^+^/MyoD^+^ double immunofluorescence staining. Here we found that very few MyoD^+^ cells expressed Ki67, and in general there were almost no Ki67 expressing cells in the tissue (Table 3 and Figures 4(j)–4(l)).

**Figure 5:**
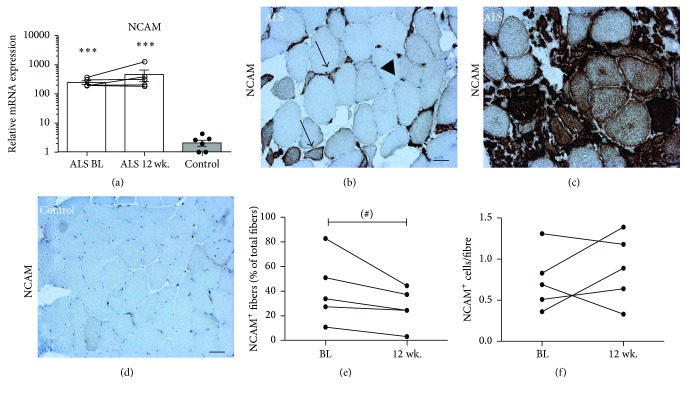
NCAM in ALS. (a) NCAM mRNA transcript in ALS during disease progression compared to controls. (b and c) Immunohistochemical staining for NCAM (blue) showed positive fibres (arrows) and cells (arrowheads) in ALS, but no staining except for a few positive satellite cells in controls (d). Stereological analysis of (e) NCAM^+^ fibres (^#^ *p* = 0.078) and (f) NCAM^+^ cells. Asterisk indicates significant difference from controls; ^*∗∗∗*^ *p* < 0.001. In (a) *n* = 5 for ALS and *n* = 7 for controls. In (b–f) *n* = 5 for ALS. Scale bar = 50 *μ*m.

To further evaluate myogenic changes in ALS muscle we analysed expression of NCAM. We observe that NCAM mRNA levels are highly increased in ALS compared to controls (Figure 5(a)). Immunohistochemistry for NCAM protein demonstrates NCAM expression in both single cells and individual fibres, which are mainly atrophic fibres (Figures 5(b) and 5(c)), whereas controls show a few NCAM^+^ satellite cells (Figure 5(d)). The disease progression induces a decrease in NCAM^+^ fibres for all ALS subjects, which trends to reach statistical significance (*p* = 0.078), but no changes in NCAM^+^ cells are observed (Figures 5(e) and 5(f)).

### 3.4. Alterations in myomiRs, Cell Cycle Regulators, and Inflammation in ALS

**Figure 6:**
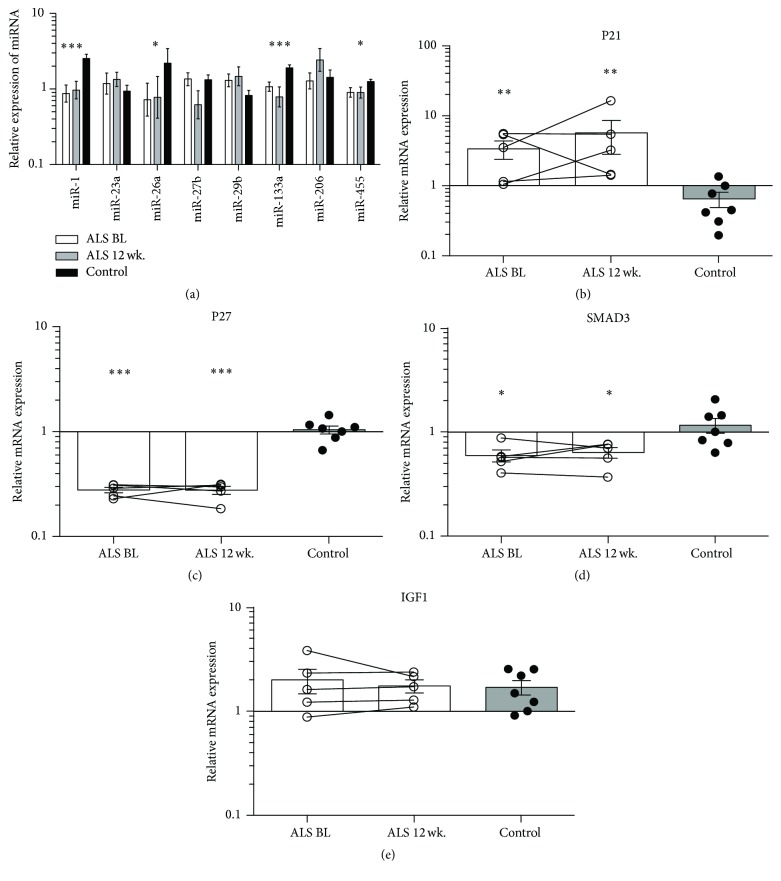
Cell cycle regulation and muscle specific microRNAs in ALS patients. (a) Expression of miR-1, miR-133a, miR-206, and miR-455 in ALS patients compared to controls. (b–e) mRNA transcript in ALS during disease progression compared to controls for (b) p21, (c) p27, (d) SMAD3, and (e) IGF-1. *n* = 5 for ALS samples and *n* = 7 for controls. Asterisks indicate significant differences from controls; ^*∗*^ *p* < 0.05, ^*∗∗*^ *p* < 0.01, and ^*∗∗∗*^ *p* < 0.001.

**Figure 7:**
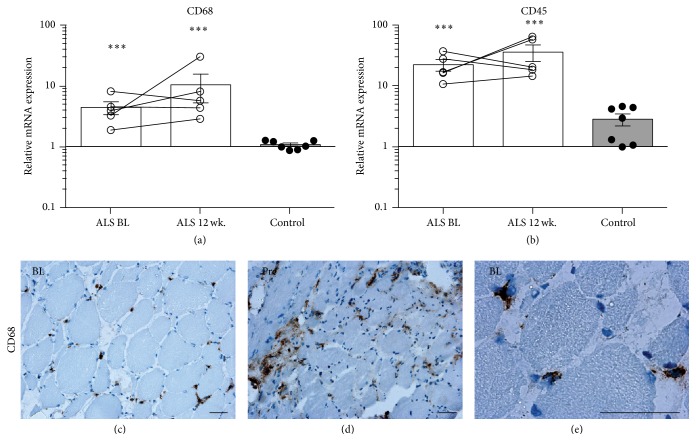
Active tissue inflammation to assist in cellular repair. (a and b) Expression levels of CD68 and CD45 mRNA. (c–e) Representative images of tissue macrophages at both BL and 12 wk. time points with a localized distribution in the interstitium between muscle cells. In (a and b) *n* = 5 for ALS and *n* = 7 for controls. In (c–e) *n* = 5 for ALS. Asterisks indicate significant differences from controls; ^*∗∗∗*^ *p* < 0.001. Scale bar = 50 *μ*m.

Skeletal muscle specific microRNAs (myomiRs) 1, 26a, and 133a along with miR-455 are reduced in ALS compared to controls (Figure 6(a)); however, disease progression does not induce further change between BL and 12 wk. mRNA expression of cyclin-dependent kinase inhibitor 1A (p21) is induced, while cyclin-dependent kinase inhibitor 1B (p27) and mothers against decapentaplegic homolog 3 (SMAD3) mRNA transcripts are reduced compared with controls (Figures 6(b)–6(d)). Insulin-like growth factor 1 (IGF1), an inducer of muscle tissue growth, is not affected in ALS compared to controls (Figure 6(e)). Both mRNA levels of CD68 and CD45 along with protein expression of CD68 demonstrated higher levels of inflammatory cells in skeletal muscle of ALS patients compared to controls (Figure 7). The CD68^+^ cells were localized mainly in the connective tissue between muscle fibres.

## 4. Discussion

In this paper we describe the degenerative and regenerative processes in skeletal muscle of ALS patients over a short-term period to observe disease development and progression. Moreover, we compare the skeletal muscle of ALS patients to that of healthy, age-matched muscle. We find morphological changes in ALS muscle indicative of active denervation and reinnervation along with a reduction in CSA, yet none of the examined mRNA markers change significantly within the 12-week time period. However, we demonstrate differences between ALS and controls in pathways controlling skeletal muscle homeostasis, cytoskeletal and regenerative markers, neurodegenerative factors, myogenic factors, cell cycle determinants, and inflammatory markers.

Upper and lower motor neuron injuries induce different morphological changes, revealing a preferential type II fibre atrophy and type I hypertrophy and muscle fibre group atrophy and fibre type grouping, respectively [20]. Previous studies have evaluated muscle morphometry in ALS patients [21] and neuromuscular disorders [22] and described characteristic features as a mix of the above with muscle fibre atrophy and hypertrophy and a large degree of fibre type grouping, which is in line with our findings. Our results demonstrate a reduction in CSA from BL to 12 wk. and a large variability in fibre CSA ranging from below 100 *μ*m^2^ to over 18.000 *μ*m^2^ indicating an active remodelling process with atrophy of some fibres and compensatory hypertrophy in others, the latter in order to overcome the loss of muscle fibres. The fact that we do not find a significant increase in hypertrophied fibres is likely a matter of lack of power, as we do see a tendency for an increase. Higher levels in *α*-actin mRNA levels compared to controls further underline this, as active remodelling leads to an activation of the *α*-actin promoter [19].

In line with this, our data show substantially elevated NCAM mRNA levels compared to controls, and high muscle fibre NCAM protein expression also suggests that regeneration is activated [23]. NCAM^+^ fibres in ALS can represent both denervated and regenerating fibres, and the observed decrease in NCAM^+^ fibres from BL to 12 wk. could be a result of either an exhausted or an impaired regeneration. It must be kept in mind that it is the number of NCAM^+^ fibres that have been evaluated, rather than the expression level of each fibre, which might change over time.

Denervated, atrophic myofibres tend to become angular and eventually result in “nuclear clumps” with little or no cytoplasm left [24], while new myogenic fibres are small and round [25]. We find that NCAM^+^ fibres in ALS patients are mainly small and angular, which suggest that these are denervated fibres rather than newly formed, regenerating fibres.

As a measure of the myogenic potential we evaluated the number of Pax7^+^ satellite cells in ALS skeletal muscle. Previous studies have established satellite cell numbers of young and old individuals to be around 0.05 Pax7^+^ cells/fibre [26] and around the same magnitude in ALS patients [27]. Our data of Pax7^+^ cells/fibre is in line with previous findings [26], indicating that satellite cells are present for potential activation [28]. We therefore performed double stainings for MyoD and Pax7 to evaluate the actual activation status of the Pax7^+^ cells present in ALS muscle. We find that very few Pax7^+^ cells express MyoD, suggesting that the satellite cells are in a nonactive, resting state and are not activated. However, since we find the presence of many MyoD^+^/Ki67^−^ cells, this points out that there has been activation of satellite cells to regenerate the muscle; however, this process has somehow been halted or distorted due to the ALS pathology.

The higher expression levels of MYOD1 and MYOG mRNA in ALS muscle could indicate an activated differentiation process. When analysing expression of MyoD on protein level we observe a high amount of MyoD^+^ cells in general, thus corresponding to the increased expression on mRNA level as well. A double staining for MyoD and the proliferation marker Ki67 reveals that only few of the MyoD^+^ cells are in an active proliferative state, suggesting that the activated muscle cells are kept in a MyoD^+^ postmitotic state. Whether this is due to a long differentiation period or the cells are arrested at this stage needs further investigation. However, it is known that both MYOD1 and MYOG are regulated by electrical stimulation [29]; thus the findings on changes in MyoD on both mRNA and protein level might reflect the changed neural activity in these patients. The observation of reduced early myogenic markers in combination with increased late myogenic markers probably reflects the disease pattern in skeletal muscle of ALS patients.

Denervation is one of the earliest signs of ALS and in line with this the denervation-induced markers CHRNA1, GDNF, and CDK5 are all highly expressed in the ALS patients as we expected. The sensitive nature and large increase of CHRNA1, GDNF, and CDK5 in ALS muscle allow these to be potential biomarkers of ALS disease development and progression.

In the present study we also investigated the mechanisms of cell cycle progression and skeletal muscle growth to evaluate if disturbances in the expression of myomiRs or cell cycle regulators could influence the remodelling and regeneration capacity of ALS skeletal muscle. MicroRNAs are tight regulators of cell fate and tissue homeostasis [30], and our results show that miR-1, miR-133a, and miR-206 are all reduced in ALS muscle suggesting that both myogenic proliferation and differentiation might be altered. Inhibition of miR-1 and miR-206 blocks the downregulation of most targets in differentiating cells, thus indicating that they are required for muscle differentiation [31], while miR-133 expression is attenuated during the early stage of muscle regeneration and represses myoblast proliferation [32]. MiR-206 has also been identified as an important player in the repair of the NMJ following nerve injury [33], and one study has identified miR-206 as a potential disease marker in SOD-1 mice and ALS patients [34]. Thus miRs appear to be important players in ALS disease progression and muscle restoration.

Multiple studies have identified interactions between miR-1 and IGF-1 showing an inverse relationship between these [35, 36]. We observed a reduction in miR-1 combined with no change in IGF-1 mRNA, which may be due to an insufficient reduction of miR-1, or the interactions between miR-1 and IGF-1 do not play a role in ALS.

We find that p21 mRNA expression is induced in the ALS patients, suggesting that myogenic cells have entered the differentiation and maturation process and thus no longer proliferate [37–39]. This is supported by our findings of MyoD^+^/Ki67^−^ cells. Likewise, our data show a reduction in p27 mRNA in ALS patients compared to controls [40, 41], which is in line with our findings of the presence of primarily nonactivated satellite cells.

The present study has several limitations. The small number of ALS patients studied here and the heterogeneous disease progression and clinical presentation seen in ALS patients in general limit our ability to generalise our findings to larger populations of ALS patients. However, a major strength of the present study is the longitudinal collection of muscle biopsies, which is unique in ALS patients.

Altogether, our results suggest that activation, proliferation, and differentiation of skeletal muscle stem cells are affected and possibly halted in ALS patients and there is therefore a generally insufficient myogenesis that cannot overcome the denervation-induced wasting. It would therefore be necessary to understand which mechanisms are affected in order to impact on the myogenic program if we are to diminish disease progression and skeletal muscle atrophy in ALS patients in the future.

## Supplementary Material

Table S1: The gene names, primers sequences (Access ID numbers) and method of analysis for the mRNA transcripts investigated in the present study.

## Figures and Tables

**Table 1:** Clinical characteristics of ALS patients included in the study.

Subject	Age	Diagnosed	Disease onset	Ambulation		ALSFRS-R	
	Years	Years		BL	12 wk.	BL	12 wk.
1	68	<1	Spinal	Walk ua	Walk ua	42	41
2	47	<1	Spinal	Walk ua	Walk ww	40	38
3	65	<1	Bulbar	Walk ua	Walk ua	38	36
4	69	15	Spinal	Walk wb	Walk wb	38	38
5	57	<1	Spinal	Walk ua	Walk ww	43	40

Ua: unassisted; wb: with ankle brace; ww: with walker. ALSFRS-R: ALS functional rating scale-revised.

**Table 2:** Fibre type distribution in ALS muscle.

	BL	12 wk.
Total fibres (mean)	300.8 ± 83.5	207 ± 38
Type I (%)	52.5 ± 6.3	54.9 ± 9.3
Type II (%)	42.7 ± 15	40.3 ± 7.5
Mixed (%)	4.9 ± 2.0	4.8 ± 1.5
CSA type I (*μ*m^2^)	3480 ± 375	3116 ± 763
CSA type II (*μ*m^2^)	3998 ± 591	3596 ± 1159
CSA mixed (*μ*m^2^)	5807 ± 679	3461 ± 1040^#^

No significant differences are observed for any parameters except for the cross-sectional area (CSA) of mixed fibres, which is reduced at 12 wk. compared to BL (^#^ *p* < 0.05).

**Table 3:** Analysis of activation status of satellite cells in ALS.

Subject	Pax7^+^	Pax7^+^/fibre	Pax7^+^/MyoD^−^	Pax7^+^/MyoD^+^	Ki67^+^/MyoD^−^
BL					
1	13	0.071	12	2	1
2	32	0.066	30	2	2
3	26	0.074	23	3	0
4	11	0.092	11	0	1
5	22	0.068	22	0	0
Mean ± SEM	20.8 ± 3.9	0.074 ± 0.005	19.6 ± 3.6	1.4 ± 0.6	0.8 ± 0.37

12 wk.					
1	10	0.057	9	1	0
2	10	0.067	9	1	0
3	21	0.088	21	0	1
4	23	0.099	23	0	0
5	25	0.091	25	0	0
Mean ± SEM	17.8 ± 3.25	0.080 ± 0.01	17.4 ± 3.5	0.4 ± 0.2	0.2 ± 0.2

Satellite cells/myoblast in BL and 12 wk. patient samples were stained for expression of Pax7, MyoD, and Ki67 using immunofluorescence and evaluated by counting. The number of identified cells in each section is noted.
