# Supplementary material for: Skeletal Muscle Remodelling as a Function of Disease Progression in Amyotrophic Lateral Sclerosis
Source: Biomed Res Int. 2016 Apr 18;2016:5930621. doi: 10.1155/2016/5930621 (PMC4852332; doi:10.1155/2016/5930621)
Supplement: Supplementary file 1 — Table S1: The gene names, primers sequences (Access ID numbers) and method of analysis for the mRNA transcripts investigated in the present study. [file 5930621.f1.pdf]

Supporting information

Table S1: The primers used to investigate mRNA expression levels, their ID numbers, function and analysis method.

Gene name	ID	Function	Run by
MYF5	Hs00929416_g1	Early myogenic marker	96-well
PAX7	Hs00242962_m1	Early myogenic marker	96-well
CDKN1A/P21	Hs00355782_m1	Cell cycle regulator	96-well
CDKN1B/P27	Hs01597588_m1	Cell cycle regulator	96-well
SMAD3	Hs00969210_m1	Role in muscle growth	96-well
IGF1	HS01547656_m1	Role in muscle growth	96-well
RPLP0	HS99999902_m1	Reference gene	96-well
GAPDH	HS99999905_m1	Reference gene	96-well
ACTA1	Hs00559403_m1	Muscle specific actin	LDA card
CDRNA1	Hs00175578_m1	Acetylcholine receptor subunit, denervated muscle	LDA card
GDNF	Hs01931883_s1	Glial cell-derived neurotrophic factor, motor neurons	LDA card
CDK5	Hs00358991_g1	Cell division protein kinase 5, neuronal maturation	LDA card
MYOG	Hs01072232_m1	Late myogenic marker	LDA card
MYOD1	Hs02330075_g1	Late myogenic marker	LDA card
NCAM	Hs00941830_m1	Expressed in satellite cells, regenerating and denervated fibres	LDA card
CD68	Hs02836816_g1	Expressed on monocytes/macrophages	LDA card
PTPRC /CD45	Hs04189704_m1	Expressed in hematopoietic cells	LDA card
RPLP0	HS99999902_m1	Reference gene	LDA card
GAPDH	HS99999905_m1	Reference gene	LDA card
